# Scrap-SAM-CLIP: Assembling Foundation Models for Typical Shape Recognition in Scrap Classification and Rating

**DOI:** 10.3390/s26020656

**Published:** 2026-01-18

**Authors:** Guangda Bao, Wenzhi Xia, Haichuan Wang, Zhiyou Liao, Ting Wu, Yun Zhou

**Affiliations:** 1 School of Metallurgical Engineering, Anhui University of Technology, Ma’anshan 243032, China; bgd98@ahut.edu.cn (G.B.); xwz423628@163.com (W.X.); which@ahut.edu.cn (H.W.); lzhy@ahut.edu.cn (Z.L.); 2 Key Laboratory of Metallurgical Emission Reduction & Resource Recycling (Ministry of Education), Anhui University of Technology, Ma’anshan 243002, China

**Keywords:** scrap identification, segment anything model, contrastive language-image pre-training, fine-tuning, 3D recognition solution

## Abstract

To address the limitation of 2D methods in inferring absolute scrap dimensions from images, we propose Scrap-SAM-CLIP (SSC), a vision-language model integrating the segment anything model (SAM) and contrastive language-image pre-training in Chinese (CN-CLIP). The model enables identification of canonical scrap shapes, establishing a foundational framework for subsequent 3D reconstruction and dimensional extraction within the 3D recognition pipeline. Individual modules of SSC are fine-tuned on the self-constructed scrap dataset. For segmentation, the combined box-and-point prompt yields optimal performance among various prompting strategies. MobileSAM and SAM-HQ-Tiny serve as effective lightweight alternatives for edge deployment. Fine-tuning the SAM decoder significantly enhances robustness under noisy prompts, improving accuracy by at least 5.55% with a five-positive-points prompt and up to 15.00% with a five-positive-points-and-five-negative-points prompt. In classification, SSC achieves 95.3% accuracy, outperforming Swin Transformer V2_base by 2.9%, with t-SNE visualizations confirming superior feature learning capability. The performance advantages of SSC stem from its modular assembly strategy, enabling component-specific optimization through subtask decoupling and enhancing system interpretability. This work refines the scrap 3D identification pipeline and demonstrates the efficacy of adapted foundation models in industrial vision systems.

## 1. Introduction

In the context of deepening commitment to carbon peaking and carbon neutrality in China, scrap steel, as the only green resource capable of large-scale substitution for iron ore, has experienced a marked increase in demand. Evaluation, grading, and sorting of scrap have therefore become critical to enabling scalable industrial development. The national standard ‘Recycling iron-steel materials’ [1], promulgated in late 2020, stipulates that dimensional assessment of scrap remains predominantly based on manual visual inspection, with physical measurements employing tools such as measuring tapes or calipers reserved for exceptional cases. This approach is not only labor-intensive but also prone to subjectivity, resulting in outcomes of limited reliability.

**Figure 1:**
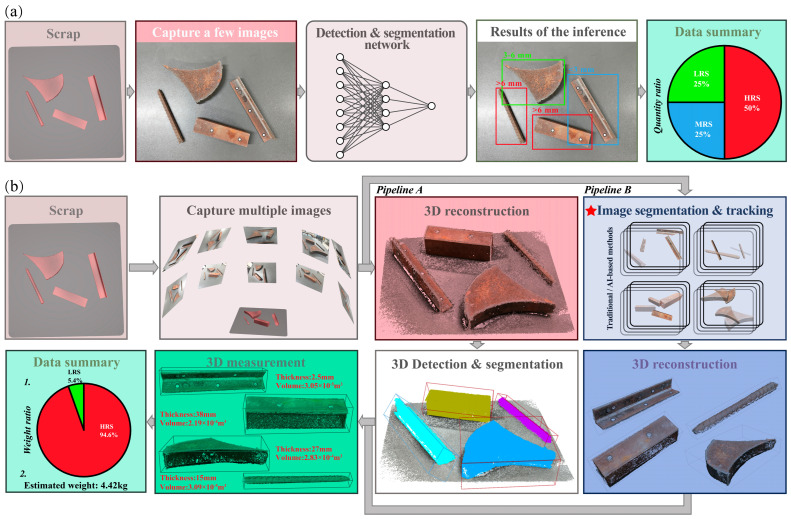
Architectural comparison of the scrap recognition frameworks. (**a**) Mainstream 2D workflow; (**b**) Proposed 3D workflow.

**Figure 2:**
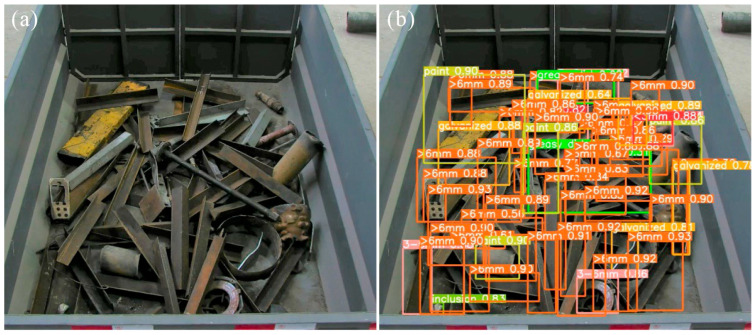
Representative dataset samples employed in conventional 2D scrap recognition frameworks. (**a**) Raw input images. (**b**) Visualized annotation masks.

Advances in computer vision have facilitated the development of research initiatives and commercial systems [2,3] for scrap classification and grading using deep learning. The 2D pipeline depicted in Figure 1a initially processes scrap images through a recognition network, subsequently conducting statistical analysis of the output to support grading decisions derived from relative proportions of distinct scrap categories. However, 2D-based approaches suffer from several inherent limitations. (i) The 2D deep learning approach primarily relies on texture and shape cues for object recognition [4]. However, scrap thickness shows no consistent correlation with these visual features; scraps of different shapes and textures may have randomly varying thicknesses. (ii) Furthermore, scrap thickness is typically several orders of magnitude smaller than its length and width, and the limited information visible in 2D images is often insufficient for algorithms to learn this dimension reliably. (iii) The system employs single-view detection, which is inherently prone to error and lacks robustness. When the same batch of scrap tumbles or slides naturally, its orientation changes; in many cases, the thickness direction is not visible, leading to significant discrepancies between repeated detections of identical material. (iv) Additionally, value assessment based solely on the quantity ratio of scrap categories often yields inaccurate results because it ignores differences in weight. Thus, the field is increasingly shifting toward 3D approaches, as exemplified by the 3D solution architecture depicted in Figure 1b. This architecture incorporates two distinct processing pipelines, differentiated primarily by the sequence of reconstruction and recognition procedures: *Pipeline A* processes reconstructed point cloud data directly, performing point cloud feature extraction and spatial pattern matching for 3D recognition of scrap. Conversely, *Pipeline B* first performs 2D recognition on multi-view images of scrap, followed by feature aggregation and reconstruction based on the 2D recognition results to reconstruct 3D features of the scrap. This study focuses on the 2D segmentation task in *Pipeline B*, which presents notable practical advantages over the 3D object detection in *Pipeline A*, chiefly owing to the latter’s substantial computational cost, more complex data processing requirements, and intricate model architectures. A critical distinction of our method from existing pipelines for 2D scrap recognition lies in the treatment of size categories. Current approaches employ fine-grained categorization (for example, 0–3 mm, 3–6 mm [2,3]), a premise that is questionable for inferring absolute 3D dimensions from 2D imagery [5], as shown in Figure 2. Our approach, by contrast, classifies scrap instances solely based on characteristic geometric shapes, thereby reducing the complexity of subsequent automated 3D dimensional analysis.

In tasks involving fixed categories under simple scenarios, closed-set detection methods are both highly efficient and reliable and thus widely adopted [6,7]. Qiu [8] and Duan [9] employed the Yolov3 algorithm to achieve target detection in scrap images, implementing modifications of the Yolo network structure to enhance the detection accuracy. By proposing a variety of deep learning algorithms that integrate different attention mechanisms, Xiao [2,3] has established a notable advantage in accuracy when compared to traditional manual quality assessment methods. Yet, the real-world scrap environment is characterized by an open-world setting, featuring diverse morphologies, varied origins, and significant regional disparities in imagery. This scenario poses a severe challenge to the generalization capabilities of such methods. Iron and steel, as the most widely used and important materials in human history, are extensively covered in existing datasets, and their shape recognition constitutes a relatively straightforward task. The large models trained on such data, leveraging robust zero-shot and few-shot generalization capabilities, are well-suited for visual perception in scrap analysis. Within computer vision, the SAM [10,11,12], built upon the ViT [13] architecture, has been trained on ultra-large-scale datasets and demonstrates exceptional zero-shot segmentation capabilities on unseen data, exhibiting superior generalization compared to traditional segmentation methods [14,15]. SAM supports multiple interactive prompt modalities, including points and bounding boxes, enabling precise specification of target locations and regions while exhibiting high generalizability. However, SAM lacks categorical information, precluding direct application in semantic or instance segmentation tasks for scrap. In comparison, the CLIP [16] model employs natural language supervision and contrastive learning to map visual and linguistic representations into a shared semantic space, thereby overcoming the limitations of traditional closed-set classification. By assembling the foundational models SAM and CLIP, we develop SSC to tackle semantic comprehension in open-world scenarios and meet fine-grained segmentation demands. This paper presents the SSC large model, which innovatively leverages strong generalization capabilities to address the diversity of scrap steel shape features, overcoming limitations of traditional recognition models that heavily rely on training data distributions and exhibit weak domain adaptability. Through lightweight fine-tuning, the model achieves robust performance with minimal annotated data, significantly reducing annotation costs while maintaining adaptability to unseen morphological variations in scrap steel.

This study fine-tunes the SSC model on a scrap dataset, employing comprehensive quantitative and qualitative experimental analyses to evaluate its capabilities in scrap perception, thereby demonstrating the potential of large-scale models for application in scrap grading and sorting.

## 2. Materials and Methods

### 2.1. SSC Model

**Figure 3:**
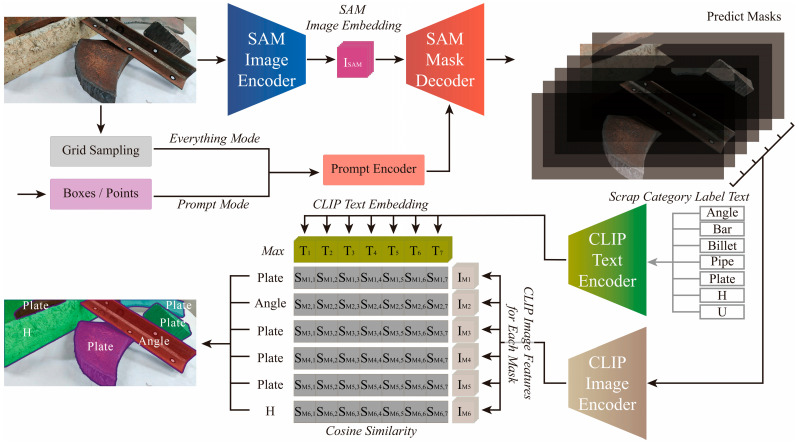
Overview of SSC.

As presented in Figure 3, SSC is a large-scale model assembled from SAM and CLIP. Unlike Visual ChatGPT [17], which employ a LLM as a controller, this approach is specifically optimized for single-task scrap recognition, resulting in a lightweight architecture, flexible workflow, and enhanced computational efficiency.

The segmentation module of the SSC architecture comprises three core components: an image encoder, a versatile prompt encoder, and an efficient mask decoder. The image encoder employs a Transformer architecture rather than a CNN, ensuring strong performance on large-scale datasets. It incorporates an advanced unsupervised pre-training method, the MAE [18], which effectively filters redundant high-level semantic information and extracts the most salient visual features, thereby significantly enhancing generalization capability. The prompt encoder converts various prompt inputs into embedding representations. Point prompts are directly embedded using positional encoding, with a learnable token distinguishing foreground from background. Bounding box prompts are represented through positional encoding derived from top-left and bottom-right coordinates. The mask decoder efficiently maps image embeddings, prompt embeddings, and an output token to the corresponding mask. Its core mechanism updates all embedding representations via prompt self-attention and bidirectional cross-attention, modeling bidirectional interactions between prompts and image embeddings. Following processing by these modules, image embeddings undergo upsampling. A multilayer perceptron then maps the output token to a dynamic linear classifier, which computes foreground probability for each image location.

**Figure 4:**
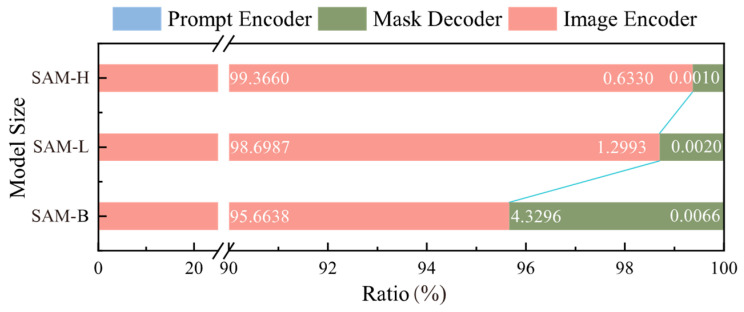
Parameter distribution across components in SAM. The parameter count of the Promote Encoder is extremely low, constituting less than 0.005% of the total, making it visually indistinct in the figure.

Both the prompt encoder and mask decoder are designed to be highly lightweight. As illustrated in Figure 4, in the three SAMs of varying scales, these components constitute a minimal proportion of parameters, with the primary computational costs concentrated in the image encoder. To extend the applicability of SAM across diverse domains, subsequent research has developed domain-adapted variants. For enhanced accuracy, HQ-SAM [19] incorporates an additional HQ-Token into the decoder alongside the original image embeddings and prompt tokens, fusing mask decoder features from SAM with both shallow and deep features from the ViT encoder to jointly exploit global semantic context and local detail information. A high-precision mask dataset (HQSeg-44K) is constructed for training, significantly enhancing segmentation accuracy. For efficiency, MobileSAM [20] employs decoupled distillation to compress the ViT-H-based image encoder into a lightweight variant, reducing image encoding time to 8 milliseconds without compromising segmentation performance. By contrast, EdgeSAM [21] distills the ViT-based image encoder into a pure CNN architecture, incorporating both the prompt encoder and mask decoder during distillation. A lightweight module is additionally incorporated to mitigate data bias from point prompt distillation, achieving a 37× speedup relative to the original SAM.

The classification module of SSC, implemented based on the CLIP model, employs the Text Encoder and Image Encoder to separately extract textual and visual features. It utilizes contrastive learning to establish correspondence between text and image representations, thereby overcoming the conventional paradigm of fixed class labels in supervised learning. Trained on a large-scale dataset encompassing diverse natural scenes, the model demonstrates exceptional generalization capabilities. CN-CLIP [22] achieves SOTA performance across multiple Chinese benchmarks through the incorporation of Chinese linguistic semantics on the text side and a two-stage training strategy. This strategy consists of an initial locked-image tuning stage followed by a contrastive tuning stage, thereby fully leveraging the inherent generalization capacity of the original CLIP framework. This study focuses on the development of an intelligent scrap identification system tailored for the domestic market. Hence, CN-CLIP, which is better suited for localized tasks, was selected as the foundational model for classification. In practice, this necessitates the conversion of English category labels into Chinese textual expressions. For the scrap segmentation results, both the category labels and the corresponding mask images are processed through text and image encoders to extract features. The optimal match between labels and masks is then determined using cosine similarity.

### 2.2. Datasets

**Figure 5:**
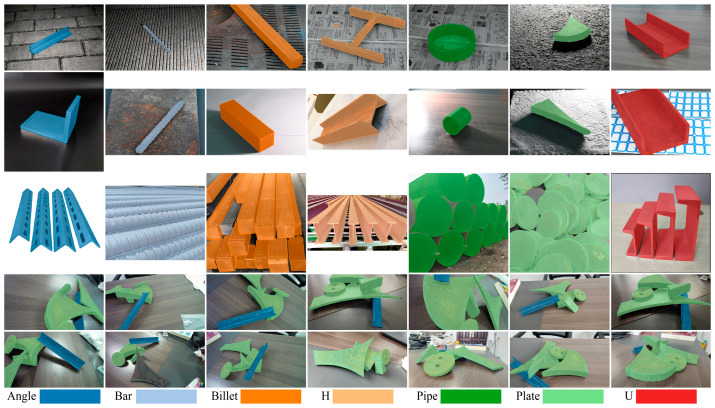
The segmentation dataset featuring representative 3D scrap shapes.

Current mainstream 2D detection approaches address fine-grained challenges particularly evident during data preparation as illustrated in Figure 2, requiring manual on-site thickness measurements of scrap using measuring tapes alongside bounding box and instance mask annotations for each object, resulting in substantial annotation complexity and high operational costs. In contrast, the 2D detection process is exclusively concerned with the canonical shapes of scrap, which are categorized into seven classes: ‘Angle’, ‘Bar’, ‘Billet’, ‘H’, ‘Pipe’, ‘Plate’ and ‘U’, as illustrated in Figure 5. This approach significantly reduces annotation costs and enables dataset expansion through the utilization of existing online scrap images. The dataset comprises 5293 images sourced from web-based imagery, steel plant, and laboratory capture, partitioned into 85% training and 15% testing subsets, totaling 17,537 annotated scrap instances.

**Figure 6:**
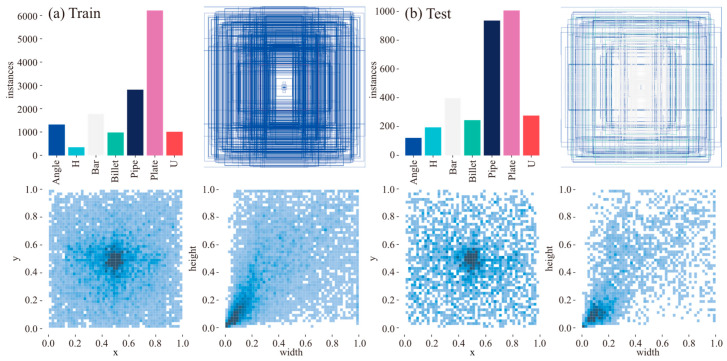
Categories, dimensions, and spatial distribution of the training (**a**) and test (**b**) datasets.

**Figure 7:**
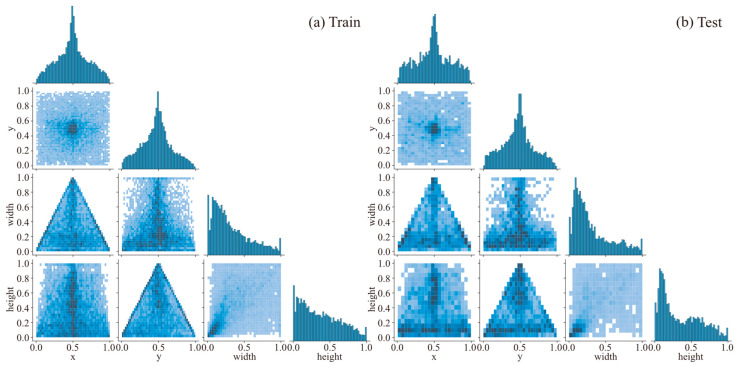
Correlation analysis of geometric attributes in the training (**a**) and test (**b**) datasets.

Figure 6 and Figure 7 provide a comprehensive analysis of the training and test set properties. Results indicate strong consistency and balance in category distribution, spatial location, and geometric characteristics between the two sets, establishing a reliable foundation for model training and evaluation. Furthermore, the scatter plot of instance width versus height reveals a distribution clustered in the lower left quadrant, indicating a high prevalence of small objects that poses a significant challenge to the segmentation capability of the SSC model. Based on the segmentation dataset, individual scrap instances were extracted to establish a classification dataset for evaluating the classification performance of the SSC model. To ensure data quality, instances with excessively small mask areas were filtered out, and images with indistinct features were manually excluded. The final classification dataset comprises 5626 scrap images, partitioned into training and testing subsets at an 85:15 ratio.

## 3. Results

All experiments were conducted on two NVIDIA RTX 4060Ti GPUs with 16 GB of VRAM each. The proposed SSC framework was implemented in Python (version 3.10.13) using PyTorch (version 2.1.2) and torchvision (version 0.16.2). For fair comparison, other classification networks were implemented using either the TIMM library (version 0.4.12) and torchvision.

### 3.1. Finetune of SSC Model

The SSC architecture is a fully decoupled model in which the SAM and CLIP modules are independently fine-tuned. This strategy ensures that each submodule maintains optimal performance on its respective tasks while reducing the complexity inherent in training a unified model.

**Figure 8:**
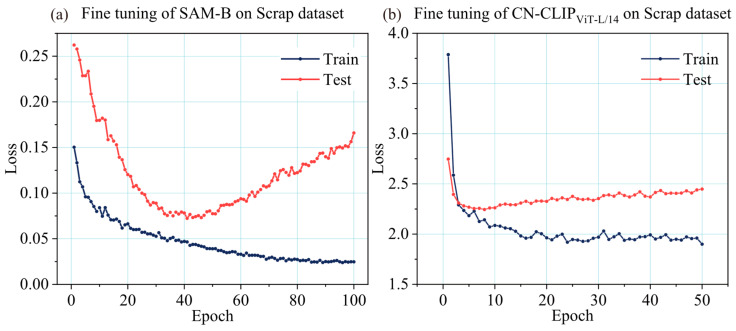
Fine-tuning loss curves of the SSC model on the scrap dataset. (**a**) Segmentation module (SAM-B). (**b**) Classification module (CN-CLIP_ViT-L/14_).

Owing to the widespread use of steel materials, which are thus well-represented in large-scale datasets such as SA-1B, their visual features have been effectively learned during pre-training. Furthermore, considering the large parameter count of the SAM image encoder and the significant computational cost of fine-tuning, this study fine-tunes only the mask decoder with all other parameters frozen. Prompt types include point prompts and box prompts. Point prompts are generated by randomly sampling foreground and background points from the annotation mask at a 2:1 ratio, while box prompts derive from the minimum bounding rectangle of the mask. To improve model generalization across both prompt types, a stochastic omission strategy is employed during training, randomly excluding either prompt type with a 50% probability. The loss function combines BCE [23] loss and DICE [24] loss to jointly optimize pixel-level classification accuracy and segmentation overlap. The AdamW [25] optimizer is used with a CosineAnnealingLR [26] scheduler, an initial learning rate of 1 × 10^−4^, and a batch size of 1. The SAM-B model undergoes 100 epochs of fine-tuning. Training error convergence curves in Figure 8a demonstrate consistent convergence, leading to the selection of weights from the 41st epoch as the final model.

The SSC classification module is implemented based on chinese-clip-vit-large-patch14-224px. On the image encoding side, it employs the same ViT-L architecture as CLIP, while on the text encoding side, it utilizes RoBERTa [27] rather than GPT-2 [28]. This design yields enhanced performance in long-text comprehension and classification tasks, and demonstrates particularly improved effectiveness in processing Chinese semantic information. During fine-tuning, we followed the official recommendations for hyperparameters: batch size of 48, initial learning rate of 1 × 10^−6^, AdamW optimizer, and a cosine learning rate scheduler. The model was initially configured to train for 500 epochs. As overfitting became evident around epoch 50, early stopping was applied. The training was halted, and the model from the first 50 epochs was saved for further use. The training error curve is presented in Figure 8b, and the weights from the 9th epoch were selected as the final fine-tuned model.

### 3.2. Evaluation of SSC Segmentation Performance

#### 3.2.1. Evaluation Criteria and Strategies for Segmentation

To evaluate the segmentation performance of the SSC model, this study employs three metrics: the DICE, the Jaccard Similarity Coefficient (JAC, also called IOU [29]), and the Hausdorff Distance (HD [30]). DICE and JAC both measure the similarity between two binary masks, with values ranging from 0 to 1, where higher values indicate greater agreement. For binary masks *A* and *B*, these metrics are defined as follows:

(1) $$\mathrm{DICE}(A,B)=\frac{2\cdot \left|A\cap B\right|}{\left|A\right|+\left|B\right|}$$

(2) $$\mathrm{JAC}(A,B)=\frac{\left|A\cap B\right|}{\left|A\cup B\right|}$$

where $\left|A\cap B\right|$ denotes the number of pixels in the intersection of masks *A* and *B*, $\left|A\right|$ and $\left|B\right|$ represent the total number of pixels in each mask, respectively, and $\left|A\cup B\right|$ indicates the number of pixels in the union of the two masks.

HD measures the maximum distance between two pointsets, typically used to quantify the discrepancy between segmentation boundaries. For two pointsets *X* and *Y*, the one-sided HD from *X* to *Y* is defined as:

(3) $$\mathrm{hd}(X,Y)=\underset{x\in X}{\max}\underset{y\in Y}{\max}\left\|x-y\right\|$$

Correspondingly, the one-sided HD from *Y* to *X* is defined as:

(4) $$\mathrm{hd}(Y,X)=\underset{y\in Y}{\max}\underset{x\in X}{\max}\left\|x-y\right\|$$

The bidirectional HD between these two sets is then:

(5) HD(X,Y)=max(hd(X,Y),hd(Y,X))

The above calculations are based on Euclidean distance to measure between point pairs.

Additionally, as SAM may generate multiple binary masks for a single input image, not all of which correspond to the target object, we employed the mask matching mechanism described in [11] to evaluate the segmentation performance under each test mode. For a given object in an image, DICE scores are computed between each predicted binary mask *P_n_* and the ground truth mask *G*. The predicted mask with the highest DICE score is selected as the matched mask *P^∗^* for subsequent segmentation evaluation. This matching process is formalized in Equation (6).

(6) $$P^{*}=\underset{P_{n}}{\mathrm{argmax}}(\{\mathrm{DICE}(P_{1},G),\;\mathrm{DICE}(P_{2},G),\;\ldots ,\;\mathrm{DICE}(P_{n},G)\})$$

SAM and its improved variants support both a fully automatic segmentation mode and a manual prompt mode. We adopted six evaluation strategies (S1–S6) based on the methodology in [11], defined as follows:

- S1: automatic everything mode;
- S2: one positive point;
- S3: five positive points;
- S4: five positive and five negative points;
- S5: one box;
- S6: one box and one positive point.

Implementation details are as described in Section 3.3 of [10].

#### 3.2.2. Segmentation Capability Under Different SAMs

**Figure 9:**
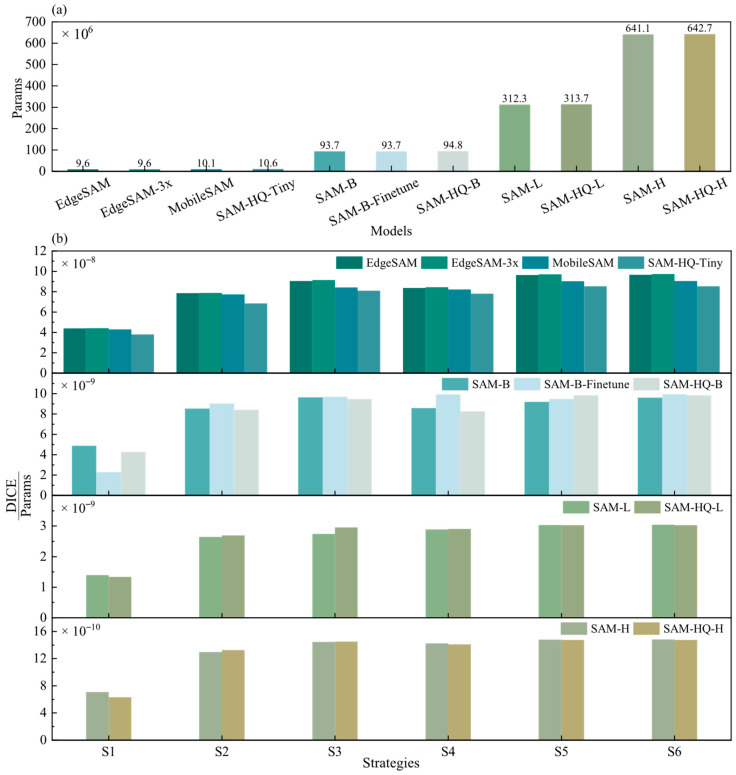
Model scale (**a**) and parameter efficiency (**b**) of SAM variants across different strategies.

**Figure 10:**
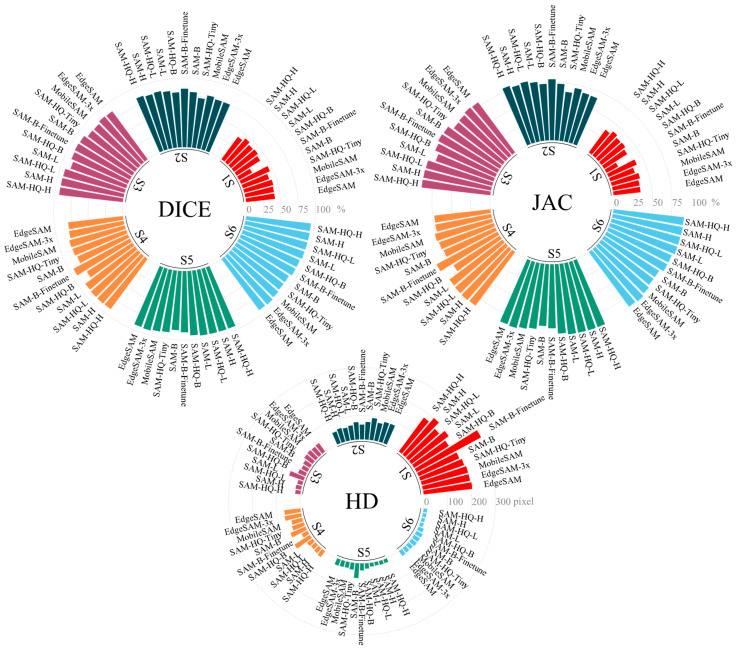
Comparison of the average performance of SAM variants under different strategies.

This section compares the segmentation performance of various SAM-based models on the scrap dataset. Figure 9 presents the efficiency metrics (DICE/Params) of different SAM variants, which are categorized into four groups: Tiny (EdgeSAM, EdgeSAM3x, MobileSAM, and SAM-HQ-Tiny), Small (SAM-B, SAM-B-Finetune, and SAM-HQ-B), Medium (SAM-L and SAM-HQ-L), and Large (SAM-H and SAM-HQ-H). Results in Figure 10 indicate that within the Tiny model group, EdgeSAM3x achieves superior efficiency and significantly enhanced segmentation accuracy under strong prompt conditions. On the other hand, MobileSAM demonstrates superior performance in weak prompt scenarios (S1: DICE 1.24%↑; S2: DICE 2.75%↑). In the Small model group, fine-tuning substantially improved model accuracy, with DICE score improvements ranging from 0.7% under strategy S1 to 12.53% under S4. A significant performance degradation was observed in the no-prompt mode after fine-tuning (S1: DICE 24.2%↓). This decline may originate from suboptimal hyperparameter settings, such as non-maximum suppression parameters and confidence thresholds, which still require further optimization. Furthermore, SAM-B demonstrates higher efficiency under weak prompt conditions, whereas SAM-HQ-B is better suited for strong prompt scenarios. In the Medium and Large model groups, the SAM-HQ series exhibits moderate improvements in point-prompt settings but shows reduced accuracy in no-prompt mode (SAM-HQ-L S1: DICE 1.48%↓; SAM-HQ-H S1: DICE 4.82%↓). Models in the Medium and Large groups demonstrate comparable performance, with larger parameter counts yielding no significant improvements. This stems from marginal gains in feature extraction capability beyond a certain model scale for scrap recognition tasks, whereas prompt quality critically determines SAM performance as a prompt-based model. For tasks prioritizing optimal performance, models in the Medium group suffice.

**Figure 11:**
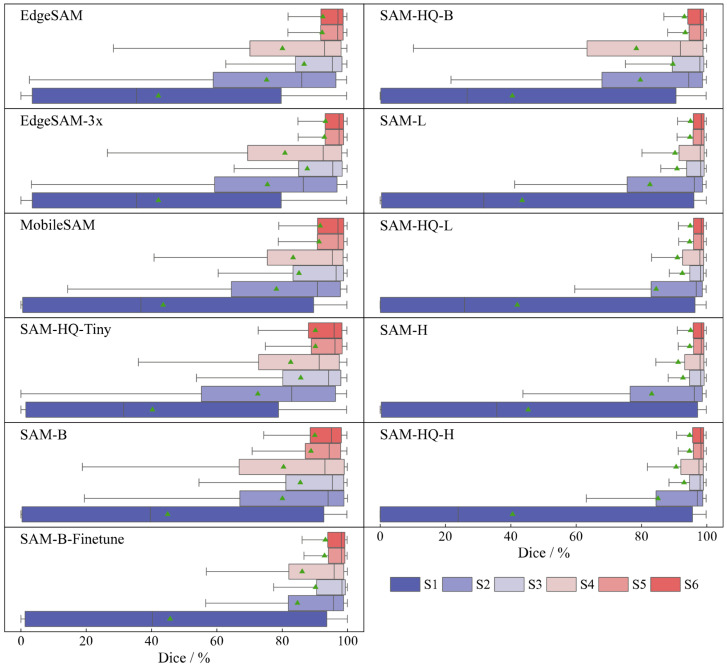
DICE performance of SAM variants on scrap under different strategies. The box plots illustrate the distribution of DICE scores, with triangles indicating the mean values.

**Figure 12:**
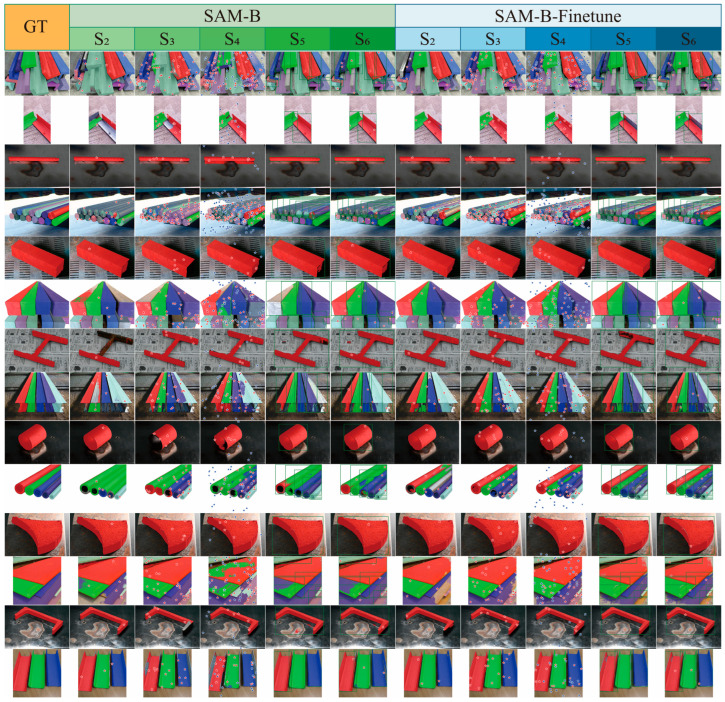
Visualization of SAM variants under different strategies on typical scrap datasets. Red and blue stars rep-resent positive and negative point prompts, respectively. The green box indicates the box prompt.

Figure 11 displays the distribution of DICE across different models using box plots. Among Tiny models, MobileSAM shows greater stability and lower variance under weak prompt conditions, whereas EdgeSAM3x achieves superior stability with strong prompts. The SAM-HQ variants exhibit improved stability relative to the original SAM under point-based prompting conditions. The fine-tuning procedure further enhances model robustness, with SAM-B-Finetune demonstrating a more compact distribution of Dice scores across all prompting modes, as illustrated in Figure 12.

#### 3.2.3. Segmentation Capability Under Different Testing Modes

This section compares the segmentation performance of various SAM variants under different evaluation strategies. Performance trends documented in Figure 10 show consistent patterns across all SAM variants throughout various testing strategies. The no-prompt mode consistently yields the lowest performance across all models. Among point-based prompt strategies (S2–S4), S3, employing one positive and one negative point, achieves the highest segmentation accuracy for SAMs. Adding more points fails to improve performance and instead degrades both accuracy and stability. This tendency likely arises from a propensity of the model to generate overly localized predictions when provided with excessive point prompts. This tendency likely arises from a propensity of the model to generate overly localized predictions when provided with excessive point prompts. For scrap with complex textures and shapes, this frequently causes the model to emphasize local features over the entire object, reducing segmentation accuracy (SAM-H S3→S4: DICE 1.52%↓; SAM-B S3 → S4: DICE 9.75%↓). Additionally, S3 exhibits a tighter distribution of DICE, reflecting enhanced stability compared to S4.

The fine-tuned model, however, exhibited the opposite trend (SAM-B-Finetune S3 → S4: DICE 2.09%↑). This enhancement demonstrates that fine-tuning facilitated a clearer perceptual understanding of scrap features within the mask decoder, resulting in more balanced learning of localized and holistic attributes. As a result, additional point prompts no longer caused the model to overemphasize localized details. The box prompt mode yielded superior accuracy and model stability. Moreover, adding an extra point prompt did not substantially enhance performance. Box prompts deliver more explicit information by defining the precise spatial location of the scrap, thereby emphasizing features within a confined region. In contrast to other methods, point prompts only indicate local features without explicit spatial constraints, which can introduce ambiguity and lead to misinterpretation.

#### 3.2.4. Inference Time Analysis of SAM

Inference time constitutes a critical metric for model performance evaluation. As presented in Table 1, we report average computational time for embedding generation, prompt encoding, and mask decoding on the scrap segmentation dataset. The results indicate that computational load during SAM inference is predominantly concentrated in the encoding phase, consistent with analysis of parameter distribution. Models within the same variant exhibit virtually identical latencies for both prompt encoding and mask decoding. While variations exist across different variants, attributable to disparities in their network architectures, all variants maintain a high level of computational efficiency. The S1 strategy demands substantial computational resources for real-time prompt encoding and mask decoding due to processing hundreds of points across the entire image and computationally intensive post-processing steps such as NMS. The decoder in SAM-HQ introduces additional computational overhead through its global-local feature fusion mechanism, leading to a moderate increase in inference time. Among lightweight models, MobileSAM achieves the shortest inference time, demonstrating viability for real-time segmentation applications.

#### 3.2.5. Evaluating the Robustness of SAM Architectures to Noisy Prompt Variations

In prior experiments, a fixed box and point selection strategy was employed to ensure experimental reproducibility. The theoretical optimal performance of SAM was evaluated through selection of mass centers and tight bounding boxes, as these are hypothesized to capture the most representative target features. In practical applications, prompts typically originate from manual input or detectors, where the provided points or bounding boxes often deviate from precise object centers or exact boundaries. Accordingly, varying degrees of positional randomness were introduced to simulate real-world implementation errors. Specifically, boxes and points were randomly shifted or expanded within pixel ranges of 0–10, 10–20, and 20–30 [11,31]. As detailed in Table 2, randomized experiments were repeated five times, with average DICE score degradation relative to baseline configurations reported. The DICE drop metric quantifies the average performance decline compared to non-perturbed reference cases.

For prompt modes, Table 2 demonstrates that increasing point prompt count from S2 to S4 reduces performance degradation and enhances model robustness. Under box prompt mode, incorporating an additional point preserves accuracy when prompts contain errors. Analysis of Figure 10 and Figure 11 indicates comparable segmentation accuracy between modes S5 and S6. Supplementary positional information improves robustness, particularly when prompts exhibit imprecision. Accordingly, for real-world applications involving automatic prompt-based detection, adopting mode S6 yields optimal segmentation performance.

Given that baseline accuracy varies across models, comparing robustness solely based on DICE reduction offers limited insight. We therefore introduce the robust accuracy (RA) metric for a comprehensive performance evaluation, defined as:

(7) $$\mathrm{RA}\;=\;\mathrm{DICE}\;-\;\bar{\mathrm{DICE}\;\mathrm{drop}}$$

where DICE represents the mean segmentation accuracy, and $\bar{\mathrm{DICE}\;\mathrm{drop}}$ denotes the average reduction in DICE measured over five experimental trials following the introduction of random prompt offsets within the ranges of 0–10, 10–20, and 20–30 pixels, respectively.

**Figure 13:**
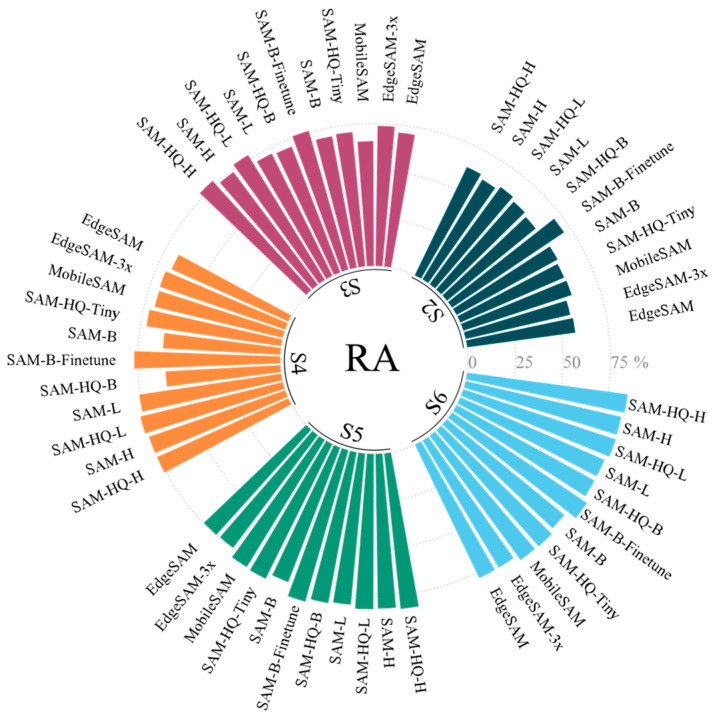
Comparison of the robust accuracy of SAM variants under different strategies.

Furthermore, Figure 13 presents the RA results for all models under diverse prompting strategies. The data demonstrate that SAM-HQ consistently attains the highest RA scores across nearly all prompt modes relative to models of equivalent scale, as evidenced by its superior overall accuracy. Among Tiny-scale architectures, MobileSAM and SAM-HQ-Tiny exhibit comparable RA performance. Moreover, fine-tuning significantly enhances model performance, enabling the fine-tuned SAM-B to match or surpass larger-scale SAM-HQ models while demonstrating particularly notable improvements under imprecise prompts, with a minimum 5.55% RA increase under the S3 strategy and a maximum 15.00% increase under the S4 strategy.

### 3.3. Evaluation of SSC Classification Performance

To rigorously assess the generalization capability of the SSC classification module compared to conventional classification models, comparative experiments were conducted. SOTA network architectures, including ConvNeXt_base [32] and Swin Transformer V2_base [33], were evaluated under two distinct training protocols: training from scratch and fine-tuning using pre-trained models. Following CN-CLIP, we use a 224 × 224-pixel input resolution wherever possible. Otherwise, the nearest compatible resolution is adopted. We employed the cross-entropy loss for optimization. The Adam optimizer was chosen because preliminary experiments showed that it achieves more stable convergence of the training loss compared to AdamW and SGD. For VGG19, which did not converge within 500 epochs when trained with Adam, we used SGD instead. An initial learning rate of 0.004 was used with a step learning rate scheduler that decayed the rate by a factor of 0.5 every 50 epochs over a total of 500 training epochs.

#### 3.3.1. Evaluation Criteria for Classification

Classification performance was assessed using four standard metrics: Accuracy, Precision, Recall, and F1 Score [34]. Accuracy quantifies the overall proportion of correctly classified instances. Precision quantifies the ability of a model to achieve correct identification of positive cases and to minimize false positive predictions. Recall measures the fraction of actual positives that are correctly detected. The F1 Score represents the harmonic mean of Precision and Recall, offering a balanced measure especially useful under class imbalance. Collectively, these metrics provide a comprehensive evaluation of the performance, reliability, and robustness of the model, enabling detailed analysis of its applicability across diverse scenarios [35]. The mathematical expressions of these metrics are provided in Equations (8)–(11):

(8) $$\mathrm{Accuracy}\;=\;\frac{TP+TN}{TP+TN+FN+FP}$$

(9) $$\mathrm{Precison}=\frac{TP}{TP+FP}$$

(10) $$\mathrm{Recall}=\frac{TP}{TP+FN}$$

(11) $$F1=2\times \frac{\mathrm{Precison}\cdot \mathrm{Recall}}{\mathrm{Precison}+\mathrm{Recall}}$$

where *TP*, *TN*, *FP*, and *FN* represent the true positives, true negatives, false positives, and false negatives, respectively.

In addition to quantitative metrics, qualitative visualization techniques provide critical insights into model behavior. Confusion matrices offer a detailed breakdown of classification results, systematically identifying patterns of misclassification. Furthermore, methods such as t-SNE [36] enable the projection of high-dimensional features into a lower-dimensional space for cluster analysis. These visualizations directly corroborate the quantitative metrics: effective classification is indicated by high values concentrated along the diagonal of the confusion matrix and by well-separated clusters in the t-SNE projection, both of which typically correspond to superior quantitative performance scores.

#### 3.3.2. Quantitative Comparison of SSC Against SOTA Models in Scrap Classification Performance

As shown in Table 3, the SSC classification module achieved Accuracy, Precision, and Recall values 2.8%, 2.7%, and 2.4% higher, respectively, than the second-best results (Swin Transformer V2_base). CN-CLIP demonstrated exceptional zero-shot classification performance, exhibiting only a 13.6% lower accuracy compared to the supervised VGG19 model. This result further underscores the necessity of fine-tuning foundation models for scrap recognition scenarios. Furthermore, traditional classification models exhibit substantially better performance when fine-tuned from pre-trained weights compared to training from scratch, with accuracy improvements ranging from 2.9% for DenseNet_201 to 15.3% for Swin Transformer V2_base. CLIP typically employs backbone architectures that are less advanced than those of newly emerging SOTAmodels, such as Swin Transformer and ConvNeXt. However, CLIP achieves superior performance, primarily because CN-CLIP is trained on a dataset significantly larger than those used for traditional SOTA classification models like ImageNet. Through the contrastive learning paradigm, it acquires strong generalization capability, delivering considerable zero-shot classification accuracy even without fine-tuning. Building on this high baseline, further fine-tuning enables CLIP to achieve its best performance.

#### 3.3.3. Visual Analysis of SSC and SOTA Models

**Figure 14:**
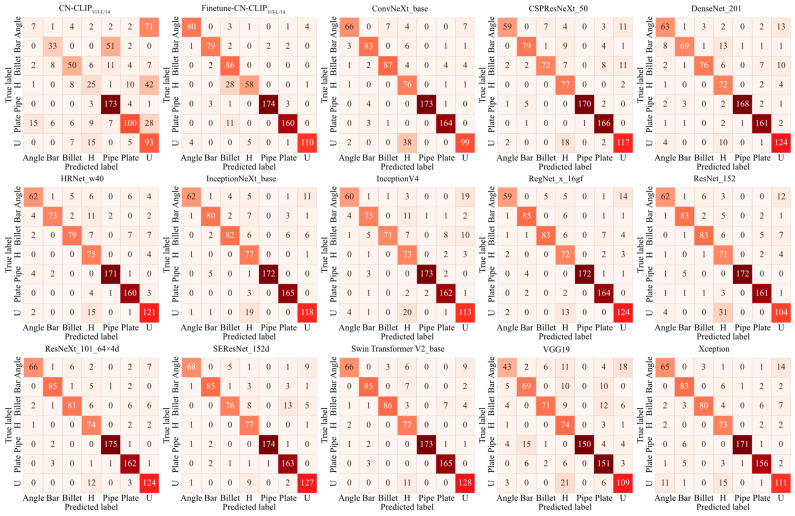
Confusion matrices of different models on the scrap classification dataset.

Figure 14 illustrates that the native CN-CLIP model demonstrates moderate classification performance for only four categories (‘Billet’, ‘Pipe’, ‘Plate’ and ‘U’), while exhibiting suboptimal accuracy for remaining categories. This limitation is attributable to the absence of corresponding labels and semantic information in the pre-training dataset. Notably, following model adaptation, classification accuracy improves significantly across all categories. Several specialized vision models misclassify ‘U’ as ‘H’ and certain ‘Angle’ instances as ‘U’, a phenomenon attributable to localized structural similarities under occlusion, which poses a significant challenge for differentiation. In comparison, the SSC framework exhibits superior performance, potentially benefiting from the robust feature extraction capabilities of the pre-trained CN-CLIP architecture.

**Figure 15:**
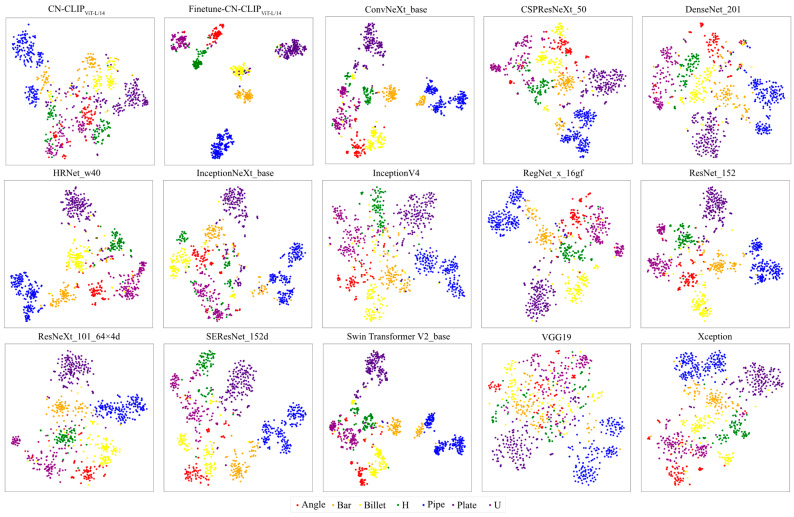
t-SNE visualization of feature representations from different models on the scrap classification dataset.

Figure 15 presents t-SNE visualizations of features extracted by various scrap classification models on the test set, with analysis conducted exclusively on visual features for both CN-CLIP and SSC. The SSC framework demonstrates superior clustering effectiveness in the feature space, exhibiting the most distinct inter-class separation boundaries. In contrast, VGG19 shows nearly indiscernible cluster boundaries, consistent with its poor classification performance observed in previous evaluations. Better-performing conventional models display clearer cluster separation with fewer outliers, aligning with their classification accuracy. Collectively, the t-SNE visualization results strongly validate the superior performance of Finetune-CN-CLIP in scrap image classification.

## 4. Discussion

Based on experimental results, the SSC large model shows strong performance in recognizing typical scrap steel shapes. For segmentation, the integrated SAM achieves a DICE score of 90% with strong prompts and improves further after fine-tuning, demonstrating powerful zero-shot capability. For classification, CN-CLIP reaches 61.6% zero-shot accuracy, which increases to 95.3% after fine-tuning, surpassing traditional state-of-the-art models. These results highlight the high generalization ability, efficiency, and cost-effectiveness of the SSC approach for handling diverse and multi-source scrap steel.

However, the segmentation model of SSC performs well with strong prompts but shows suboptimal results in the “everything” mode, which operates without prompts. In this mode, segmentation relies on uniform grid point sampling, followed by non-maximum suppression for filtering and merging. This exhaustive approach increases inference time and often causes the model to overemphasize local details, leading to excessive responses on cluttered backgrounds and incomplete masks for large objects. A similar issue appears in medical image segmentation, but the scrap steel scenario is more challenging due to frequent occlusions and object overlaps. Future work may enhance the prompt-free “everything” mode by integrating techniques such as keypoint detection or superpixel segmentation. Furthermore, extreme conditions such as occlusions and noise remain major challenges to robustness and will be addressed by building targeted datasets for quantitative evaluation.

The inference time of SSC is comparable to that of traditional models. Its assembly-based design provides good scalability and avoids using an LLM for control, eliminating significant overhead. In segmentation, original SAM and SAM-SQ are too heavy for real-time use, but lightweight variants optimized through backbone redesign, distillation, and pruning achieve similar efficiency to conventional models. The mask decoder and prompt encoder add negligible computational cost, as shown in Figure 4. For classification, SSC uses CN-CLIP with standard image encoders such as ViT-L/14 or ResNet50. The text encoder introduces a minor extra cost, which can be precomputed and cached offline for scrap steel shape recognition, making its runtime impact negligible. Therefore, the computational cost of the SSC classification module is comparable to that of traditional classification models. However, due to the modular design, image features are encoded separately for both segmentation and classification. While sharing the encoder from the segmentation stage could nearly reduce computational cost by half, this would demand significant engineering efforts in model redesign and fine-tuning, with no guarantee that the shared SAM encoder would retain high classification performance and generalization capabilities.

## 5. Conclusions

The automation and refinement of scrap classification and grading constitute a significant constraint on the green transformation of the steel industry in China. To overcome the limitations in interpretability and robustness inherent in conventional 2D scrap identification, this study proposes a novel 3D analytical framework. To avoid the unreliable fine-grained dimensional inference inherent in direct 2D image analysis, the framework integrates 2D/3D recognition with 3D reconstruction methodologies to recover precise 3D data from multi-view images, which enables accurate classification and grading. This paper investigates instance segmentation for canonical scrap shapes and proposes the SSC model, which leverages the strong generalization capability of foundation models to address the challenges of complex scrap sources and significant shape variations. Through lightweight fine-tuning, SSC achieves higher efficiency and lower computational cost compared to traditional recognition approaches.

The SSC framework utilizes an assembled architecture wherein the SAM and CLIP modules are independently fine-tuned. This design maintains specialized task performance while avoiding end-to-end optimization complexity. The segmentation module employs selective decoder fine-tuning and multi-prompt training, while the classification module uses a Chinese-optimized text encoder. Both components showed stable convergence, with optimal weights selected at epochs 41 and 9, respectively, confirming the efficacy of the decoupled training approach.

In segmentation tasks, EdgeSAM-3X performs best with precise prompts, MobileSAM offers faster inference speeds, and SAM-HQ-Tiny delivers greater robustness. Models at the L and H scales perform similarly. Box prompts achieve higher segmentation accuracy than point prompts, while the addition of an extra point prompt provides the best robustness. SAM-HQ incorporates additional parameters, yielding marginally slower inference yet improved accuracy and robustness while maintaining computational efficiency. Fine-tuning the mask decoder of SAM-B substantially improves accuracy and robustness, especially under noisy prompt conditions, with performance gains of 5% to 15% (min S3: 5.55%↑; max S4: 15.00%↑).

For the classification task, fine-tuning CN-CLIP resulted in substantial performance improvements, with the tuned model significantly outperforming all specialized vision models. It achieved a leading accuracy margin of 2.9% over the second-best model, Swin Transformer V2_base. Visual analysis further confirmed the enhanced feature extraction capability of the SSC model, as evidenced by more distinct and better separated clustering boundaries in the t-SNE projection.

Overall, the proposed SSC model achieves high accuracy and robustness in instance segmentation of typical scrap shapes through assembling and fine-tuning of the base models SAM and CLIP, thereby enhancing the pipeline for 3D scrap recognition and providing effective automatic and semi-automatic annotation tools. Compared to purely 2D inference approaches, the proposed method improves the robustness and interpretability of scrap grading and classification, thus accelerating the automation of the entire scrap steel industry chain and supporting China’s transition toward short-process steelmaking.

## 6. Future Work

Within the SSC framework, the segmentation stability of the automated ‘everything’ mode would benefit from enhancement, as its practical application remains heavily reliant on accurate prompt information. Therefore, future work should explore high-quality detection models, potentially by adapting the methodology presented in this study to leverage foundation models for scrap detection tasks, thereby improving overall recognition performance.

Furthermore, the designed Pipeline B for 3D scrap recognition relies on effective cross-view instance tracking to achieve accurate multi-view matching and provide reliable data for 3D reconstruction. While segmentation or classification errors can indeed affect reconstruction accuracy, the redundancy present in multi-view data provides a degree of robustness. Such inaccuracies can be mitigated through subsequent steps such as instance tracking, image retrieval (e.g., VLAD, BOW techniques), and image matching. By integrating methods like voting, clustering, or even graph neural networks, it is possible to filter out images or instance data with excessive errors. Given the data redundancy across multiple views, trading off a portion of the data for enhanced robustness is a worthwhile strategy.

The current 3D processing pipeline consists of multiple stages, including at least two key steps: 3D reconstruction and recognition. For the complex, high-dimensional task of extracting 3D scrap dimensions, exploring an end-to-end model may offer greater potential. However, such an end-to-end approach also faces several challenges, including but not limited to reliance on large-scale, high-quality annotated data; the complexity of model design and training; and the effective integration of multi-view geometric constraints with semantic understanding.

## Figures and Tables

**Table 1:** Inference time comparison across different SAM variants (micro seconds).

Model	Embedding	Prompt Encoding + Mask Decoding					
		S1	S2	S3	S4	S5	S6
EdgeSAM	41.278	3260.929	9.140	9.162	9.135	8.806	9.632
EdgeSAM-3x	41.446	3262.015	9.210	9.157	9.146	8.726	9.728
MobileSAM	37.263	3006.242	8.867	8.575	8.354	8.475	8.663
SAM-B	104.503	3155.644	9.016	9.020	9.038	8.574	9.449
SAM-B-Finetune	105.083	3154.891	9.113	9.132	8.945	8.633	9.520
SAM-L	211.181	3156.017	8.956	9.042	8.966	8.496	9.321
SAM-H	302.221	3155.332	9.048	9.118	9.096	8.566	9.394
SAM-HQ-Tiny	40.115	4731.128	11.024	11.029	10.952	10.497	11.226
SAM-HQ-B	94.646	4732.951	11.164	11.109	10.672	10.777	11.012
SAM-HQ-L	194.204	4730.579	10.947	10.875	11.023	10.032	10.964
SAM-HQ-H	297.400	4729.687	11.053	11.098	10.897	10.566	11.846

**Table 2:** Comparative analysis of DICE drop across SAM variants under different prompt shifting levels and testing strategies.

Model	Shift (Pixel)	DICE Drop (%)				
		S2	S3	S4	S5	S6
EdgeSAM	0–10	5.6	3.5	3.2	3.4	2.7
	10–20	19.1	16.4	13.7	18.1	14.3
	20–30	29.1	27.6	21.4	31.6	26.3
EdgeSAM-3x	0–10	6.4	2.9	2.8	3.6	2.9
	10–20	20.2	14.2	11.6	19.2	15.1
	20–30	30.6	25.0	19.1	33.3	27.7
MobileSAM	0–10	5.8	4.7	3.8	1.2	1.2
	10–20	19.4	20.9	14.9	11.6	9.3
	20–30	29.5	32.9	23.2	25.5	21.2
SAM-HQ-Tiny	0–10	0.8	2.1	0.6	0.0	−0.1
	10–20	14.2	15.5	11.6	10.0	7.5
	20–30	24.2	26.7	20.6	23.5	18.8
SAM-B	0–10	6.8	7.5	11.8	2.1	2.9
	10–20	20.6	20.7	18.9	9.2	9.5
	20–30	31.1	31.4	25.2	19.2	18.6
SAM-B-Finetune	0–10	3.2	7.3	8.8	−4.7	−0.3
	10–20	14.2	14.9	15.9	4.7	6.6
	20–30	23.9	22.9	23.9	17.9	16.9
SAM-HQ-B	0–10	7.4	6.8	10.3	2.5	2.9
	10–20	20.7	20.1	18.8	11.5	9.5
	20–30	30.6	29.4	24.8	23.8	18.6
SAM-L	0–10	6.8	-0.4	4.2	1.2	1.1
	10–20	22.5	15.0	15.2	13.6	11.1
	20–30	33.9	27.0	24.8	30.0	24.8
SAM-HQ-L	0–10	7.2	3.6	3.6	0.9	0.9
	10–20	22.7	15.9	14.4	11.6	9.3
	20–30	34.1	26.7	23.7	26.7	21.1
SAM-H	0–10	7.9	3.9	3.9	1.0	1.0
	10–20	23.4	19.6	16.3	12.0	9.5
	20–30	34.6	32.5	26.0	27.0	22.6
SAM-HQ-H	0–10	7.4	3.0	3.3	0.9	0.8
	10–20	23.4	16.2	15.7	11.0	8.1
	20–30	34.7	27.5	25.5	25.4	19.7

**Table 3:** Performance benchmarking of SSC against SOTA models on the scrap dataset.

Model	Publication	Pre-Training	Accuracy	Precision	Recall	F1Score
CN-CLIP_ViT-L/14_	arXiv2023	200 million image-text pairs (zero-shot)	61.6	60.7	53.4	52.8
SSC	-	200 million image-text pairs	**95.3** *	**94.6** *	**93.6** *	**93.8** *
ConvNeXt_base	CVPR 2022	ImageNet 1k	88.6	88.0	87.8	87.0
		From scratch	75.5	75.5	73.7	74.4
CSPResNeXt_50 [37]	CVPR 2020	ImageNet 1k	87.7	87.4	85.8	85.8
		From scratch	82.7	82.1	80.8	81.3
DenseNet_201 [38]	CVPR 2017	ImageNet 1k	86.8	86.1	84.8	84.8
		From scratch	83.9	83.2	82.2	82.6
HRNet_w40 [39]	TPAMI 2021	ImageNet 1k	87.8	87.3	86.0	85.9
		From scratch	84.8	84.3	82.6	83.3
InceptionNeXt_base [40]	CVPR 2024	ImageNet 1k	89.6	89.2	88.1	87.9
		From scratch	78.8	77.6	77.2	77.4
InceptionV4 [41]	AAAI 2017	ImageNet 1k	86.1	85.9	84.0	84.1
		From scratch	80.5	80.3	79.1	79.6
RegNet_x_16gf [42]	CVPR 2020	ImageNet 1k	89.9	89.2	88.1	88.1
		From scratch	82.4	82.0	80.5	81.0
ResNet_152 [43]	CVPR 2016	ImageNet 1k	87.2	86.3	85.8	85.5
		From scratch	80.4	80.8	78.5	79.1
ResNeXt_101_64×4d [44]	CVPR 2017	ImageNet 1k	90.9	90.4	89.4	89.5
		From scratch	86.6	86.4	85.2	85.7
SEResNet_152d [45]	CVPR 2018	ImageNet 1k	91.2	90.9	89.9	90.0
		From scratch	82.2	81.5	80.7	80.9
Swin Transformer V2_base	CVPR 2022	ImageNet 1k	92.4	91.8	91.2	91.1
		From scratch	77.1	76.9	74.6	75.5
VGG19 [46]	ICLR 2015	ImageNet 1k	79.0	78.4	77.2	76.6
		From scratch	75.2	74.9	73.9	74.3
Xception [47]	CVPR 2017	ImageNet 1k	87.6	86.4	86.5	86.1
		From scratch	83.2	83.3	81.4	82.2

* The bolded values in the table indicate the best metrics.

## Data Availability

The data presented in this study are available on request from the corresponding author. The data are not publicly available due to ongoing study.

## Abbreviations

The following abbreviations are used in this manuscript:

t-SNE	t-distributed stochastic neighbor embedding
ChatGPT	Chat generative pre-trained transformer
ViT	Vision transformer
LLM	Large language model
SGD	Stochastic gradient descent
CNN	Convolutional neural network
MAE	Masked autoencoder
SOTA	State-of-the-art
VRAM	Video random access memory
BCE	Binary cross-entropy
DICE	Dice coefficient
AdamW	Adaptive moment estimation with weight decay
CosineAnnealingLR	Cosine annealing learning rate
NMS	Non-maximum suppression
VLAD	Vector of local aggregated descriptors
BOW	Bag of words
